# Integrated mRNA and miRNA profiling in NIH/3T3 cells in response to bovine papillomavirus *E6* gene expression

**DOI:** 10.7717/peerj.7442

**Published:** 2019-08-02

**Authors:** Feng Pang, Mengmeng Zhang, Guohua Li, Zhenxing Zhang, Haifeng Huang, Baobao Li, Chengqiang Wang, Xiaohong Yang, Yiying Zheng, Qi An, Luyin Zhang, Li Du, Fengyang Wang

**Affiliations:** College of Animal Science and Technology, Hainan Key Lab of Tropical Animal Reproduction & Breeding and Epidemic Disease Research, Hainan University, Haikou, China

**Keywords:** Bovine papillomavirus, RNA sequencing, miRNA sequencing, E6, Immune response, miRNA-gene network

## Abstract

Delta bovine papillomaviruses (δBPVs) mainly infect cattle and cause fibropapillomas. δBPVs encode three oncogenes, *E5*,* E6* and *E7*. The effect of *E6* on microRNA (miRNA) and mRNA expression profiles is not well characterized. In this study, RNA sequencing and small RNA sequencing were used to explore alterations in mRNAs and miRNAs in *E6* over-expressing NIH/3T3 cells (NH-E6) compared with control cells (NH-GFP). We found that 350 genes (181 upregulated and 169 downregulated) and 54 miRNAs (26 upregulated and 28 downregulated) were differentially expressed (DE) following *E6* expression. The top 20 significantly enriched GO terms in “biological process” included inflammatory response, innate immune response, immune response, immune system process, positive regulation of apoptotic process, cell adhesion, and angiogenesis. We constructed a potential miRNA-gene regulatory network from the differentially expressed genes (DEGs) and DE miRNAs. Finally, we selected 19 immune-response related DEGs and 11 DE miRNAs for qPCR validation. Of these, upregulation of 12 genes,* Ccl2*,* Ccl7*,* Cxcl1*,* Cxcl5*,* Tlr2*,* Nfkbia*,* Fas*,* Il1rl1*,* Ltbp1*,* Rab32*, and *Zc3h12a*,* Dclk1* and downregulation of four genes, *Agtr2*, *Ptx3*, *Sfrp1*, and *Thbs1* were confirmed. *Ccl2*,* Ccl7*,* Cxcl1* and* Cxcl5* were upregulated more than ten-fold in NH-E6 compared with NH-GFP. Also, upregulation of three miRNAs, *mmu-miR-129-2-3p*, *mmu-miR-149-5p-R-2* and *mmu-miR-222-3p*, and downregulation of five miRNAs, *mmu-miR-582-3p-R+1*, *mmu-miR-582-5p*, *mmu-miR-708-3p*, *mmu-miR-708-5p* and *mmu-miR-1197-3p*, were confirmed. Our study describes changes in both mRNA and miRNA profiles in response to BPV *E6* expression, providing new insights into BPV *E6* oncogene functions.

## Introduction

Papillomaviruses (PVs) are double stranded circular DNA viruses that possess several early open reading frames (ORFs) and two late ORFs (Bernard et al., 2010; Munday, 2014). PV genomes are approximately 8000 nucleotides in length (Bernard et al., 2010). Bovine papillomaviruses (BPVs) consist of fourteen types that can be classified into four genera: Deltapapillomavirus (δPV), Xipapillomavirus (xPV), Epsilonpapillomavirus (εPV) and Dyoxipapillomavirus (Roperto et al., 2016). Among these, BPV-1, -2, -13 and -14 belong to the δPV genus and are commonly termed fibropapillomaviruses. They can infect the epithelium and also the underlying derma causing fibropapillomas of the skin and udders, and urinary bladder cancer in cows (Lubna & Saveria, 2010; Shafti-Keramat et al., 2009). The genome is divided into three regions: the early genes (*E5*, *E6*, *E7*) encoding non-structural proteins, the late genes (*L1* and *L2*, encoding viral capsid proteins) and the long control region (LCR) responsible for viral replication and transcription regulation (Lubna & Saveria, 2010).

Distinct and typical focus formation could be induced by BPV in two mouse cell lines (NIH/3T3 and C127). Therefore, NIH/3T3 and C127 cells are often used as cell models for cell transformation research in bovine papillomavirus especially in fibropapillomaviruses (Dvoretzky et al., 1980; OBrien & Campo, 1998; Suprynowicz et al., 2005; Wade, Brimer & Pol, 2008). In BPV-1, E5 is the major transforming oncoprotein followed by E6, and E7 (Jackson et al., 1991; Lubna & Saveria, 2010). The BPV-1 *E6* gene encodes a protein of 137 amino acid residues. BPV E6 can induce cell transformation by binding to the focal adhesion protein, paxillin (Tong et al., 1997; Tong & Howley, 1997). E6 can bind to several LD motif (LDXLLXXL) repeats on paxillin and the first LD repeat is the most important for binding E6. The binding between E6 and paxillin can suppress paxillin interacting with multiple cellular proteins, including focal adhesion kinase (FAK) and vinculin (Tong et al., 1997; Tong & Howley, 1997).

MicroRNAs (miRNAs) are a large class of endogenous, non-coding, small RNAs of 22–26 nucleotides. miRNAs are found in animals, plants and even in some viruses (Krol & Loedige, 2010; Sébastien et al., 2004). They participate in multiple biological processes, including cell proliferation, apoptosis and cancer (Cheng et al., 2005; Murakami et al., 2006). Moreover, accumulating evidence indicates that miRNAs play crucial roles in regulating viral replication by targeting the virus genome or cellular proteins (Chen et al., 2017; Kumar et al., 2018; Tang et al., 2016). RNA sequencing is a powerful tool to identify differentially expressed genes in response to viral infection. Yuan et al. (2008) performed transcriptional analysis of equine fibroblasts transformed by BPV-1 to identify genes associated with BPV-1 infection. They found that DEGs were significantly enriched in multiple GO terms including “Cell adhesion”, “Cell proliferation” and “Inflammation and immunity”. However, whether BPV E6, influences host cell miRNA and mRNA expression profiles remains largely unknown.

In the present study, we constructed stable NH-E6 and NH-GFP cells expressing BPV E6 and GFP or GFP alone, respectively. We then performed small RNA sequencing and RNA sequencing to explore alterations in miRNA and gene expression in NH-E6 cells compared with and NH-GFP cells. Our findings provide new insights into the functions of BPV *E6* in the viral life cycle.

## Materials and Methods

### Cell culture

NIH/3T3 and HEK293T cells were purchased from the Cell Bank of the Chinese Academy of Sciences (Shanghai, China). They were cultured in DMEM medium supplemented with 10% fetal bovine serum (FBS; Grand Island, NY, USA), 100 U/ml penicillin and 100 mg/ml streptomycin (Grand Island, NY, USA) at 37 °C in a 5% CO_2_ incubator.

### *E6* codon optimization

To increase the expression level of E6, codon use in the original *E6* gene from BPV (BPV-1, Genbank accession no. X02346; BPV-2, accession no. M20219; BPV-13, accession no. KM258443) was optimized for Mus musculus to avoid rare or low-usage codons (Sangon Biotech, Shanghai, China). The optimized *E6* sequence was synthesized and ligated into pUCK plasmid for Sanger sequencing. Alignment analysis of the original *E6* from BPV-1, -2,-13 (Hainan strain) and the optimized BPV *E6* was presented in Fig. S1.

### Lentivirus packaging

The optimized *E6* fragment was cloned into the *Asis* I and *Mlu* I sites of lentiviral plasmid pLent-EF1a-FH-CMV-GFP-P2A-Puro (Vigenebio, Shandong, China). The C-terminus of *E6* was fused with a flag-6 ×his tag (Fig. S2). This recombinant plasmid pLent-EF1a-E6-FH-CMV-GFP-P2A-Puro and the control plasmid were co-transfected with PMD2G and PSPAX2 packaging plasmids into HEK293T cells, respectively. After 72 h, supernatant was collected for purification by ultracentrifugation. Purified recombinant lentivirus and control lentivirus with a high-titer of 1.0 × 10^8^ TU/ml (transducing units/ milliliter) were collected and stored at −70 °C.

**Table 1:** List of qPCR primers for DEGs validation.

**Gene symbol**	**Primer sequence(5′-3′)**
***Ccl2***	F:CAGGTCCCTGTCATGCTTCT
******	R:GTGGGGCGTTAACTGCATCT
***Ccl7***	F:CCACATGCTGCTATGTCAAGA
******	R:ACACCGACTACTGGTGATCCT
***Cxcl1***	F: ACTGCACCCAAACCGAAGTC
******	R:TGGGGACACCTTTTAGCATCTT
***Cxcl5***	F:GCACTCGCAGTGGAAAGAAC
******	R:CGTGGGTGGAGAGAATCAGC
*** Il1rl1***	F:AAAATTCTATGATGGGCGGGT
******	R:ATGGTGTGTTCACTAGGCGG
***Ltbp1***	F:TGGACGACCCCTAGCAATGA
******	R:AAAGGCCCCTCAAGGAAGTG
***Tlr2***	F:TCTAAAGTCGATCCGCGACAT
******	R:CTACGGGCAGTGGTGAAAACT
***Bcl10***	F:CTTCAAGTAGAAAACGGGCTGG
******	R:GCACCTAGAGAGGTTGTTGGT
***Nfkbia***	F:CGAGACTTTCGAGGAAATACCC
******	R:GTCTGCGTCAAGACTGCTACA
***Fas***	F:TGCTGGCTCACAGTTAAGAGTT
******	R: ACTCCTTCCCTTCTGTGCAT
***Dclk1***	F:GTCAAGACCACCTCAGCCTC
******	R:ATGATGGTGACCAGCTTGGG
***Rab32***	F:CGTGGGTAAGACGAGCATCA
******	R:GTTGCCAAACCGTTCCTGTC
***Trim35***	F:AACACAAGAGCCGAAAACGC
******	R:AAGCTGAAGGGCACAGACTC
***Agtr2***	F:TGCTCTGACCTGGATGGGTA
******	R:AGCTGTTGGTGAATCCCAGG
***Ptx3***	F:CGTGCATCCTGTGAGACCAA
******	R:TAGGGGTTCCACTTTGTGCC
***Thbs1***	F:CAATTTTCAGGGGGTGCTGC
******	R:CCGTTCACCACGTTGTTGTC
***Sfrp1***	F:GCTGCTCAACAAGAACTGCC
******	R:TACCTTGGGGCTTAGAGGCT
***Rab10***	F:GGCAAGACCTGCGTCCTTTT
******	R:GTGATGGTGTGAAATCGCTCC
***Zc3h12a***	F:ACGAAGCCTGTCCAAGAATCC
	R:AGTAGGGGCCTCTTTAGCCAC
***Gapdh***	F: TGTGTCCGTCGTGGATCTGA
	R: CCTGCTTCACCACCTTCTTGA

### Generation of NH-E6 and NH-GFP stable cell lines

When NIH/3T3 cells seeded in a 24-well plate reached approximately 50% confluency, they were infected with recombinant lentivirus or negative control lentivirus at a multiplicity of infection (MOI) of 50. After three days, cells were subcultured and selected for puromycin resistance (Solarbio, Beijing, China) at 1 µg/mL. One week later, we acquired a polyclonal NH-GFP stable cell line and a polyclonal NH-E6 stable cell line. An IX71 fluorescence microscope (Olympus, Tokyo, Japan) was used to observe the number of cells stably expressing GFP in NH-GFP and NH-E6 stable cells. Total RNA was extracted from a dish of NIH/3T3, NH-GFP and NH-E6 cells, respectively using an Animal Tissue RNA Purification Kit (Product #TRK-1002; LC Sciences, Hangzhou, China). Then RT-PCR was performed using a HiScript II One Step RT-PCR Kit (Vazyme, Nanjing, China) with optimized E6 primers (5′-3′):E6-F:ATGGACCTTCAGTCCTTCAGC; E6-R:TCACGGATACTTAGACCTTGAGCC and GAPDH primers (Table 1) following the manufacturer’s procedures. Approximately 60 µg cellular protein from NH-GFP and NH-E6 cells were used for western blot (WB) analysis. A mouse monoclonal anti-FLAG M2 primary antibody 1:2000 (Sigma-Aldrich, Shanghai, China) and a goat anti-mouse IgG-HRP secondary antibody 1:5000 (Santa Cruz Biotechnology, Santa Cruz, CA, USA) were used to detect E6-flag. A goat polyclonal Glyceraldehyde-3-phosphate dehydrogenase (GAPDH) primary antibody (Santa Cruz Biotechnology, Santa Cruz, CA, USA) 1:2000, and a HRP-conjugated rabbit anti-goat IgG (Boster Biotechnology company, Wuhan, China) 1:5000 were used to detect the internal control GAPDH.

### RNA isolation and RNA-seq

Three total RNA isolations from a polyclonal NH-GFP /NH-E6 stable cell line were prepared respectively using an Animal Tissue RNA Purification Kit (Product #TRK-1002; LC Sciences, Hangzhou, China).

Poly (A) RNA is purified from total RNA (2 µg) using poly-T oligo-attached magnetic beads. Following purification, the mRNA is fragmented into small pieces. Then the cleaved RNA fragments were reverse-transcribed to create the cDNA library using the mRNASeq sample preparation kit (Illumina, San Diego, USA), the average insert size for the paired-end libraries was 300 bp (±50 bp). Then we performed the paired-end sequencing on an Illumina Hiseq4000 platform (LC Sciences, Hangzhou, China). The raw reads with low quality were removed to acquire the valid reads, which were aligned to UCSC (http://genome.ucsc.edu/) Mus musculus reference genome (Accession ID: GCA_000001305.2) using HISAT package. The mapped reads of each sample were assembled using StringTie. StringTie and edgeR was used to estimate the expression levels of all transcripts. StringTie was used to evaluate expression level for mRNAs by calculating FPKM (fragments per kilobase of exon per million reads mapped). Differentially expressed mRNAs and genes were selected with fold change ≥ 2 or fold change ≤ 0.5 and with statistical significance (*p* ≤ 0.05) by R package. The raw and processed data have been deposited into the Gene Expression Omnibus database (https://www.ncbi.nlm.nih.gov/geo/) under accession number GSE129593.

### miRNA sequencing

In parallel, the same total RNA was used for small RNA sequencing using an Illumina HiSeq 2500 platform from LC Sciences (Hangzhou, China). Raw reads were subjected to an in-house program, ACGT101-miR (LC Sciences, Houston, TX, USA) to remove adapter dimers, junk, low complexity, common RNA families (rRNA, tRNA, snRNA, snoRNA) and repeats. Subsequently, unique sequences with length in 18 ∼ 26 nucleotide were mapped to Mus musclus precursors in miRBase 22.0 by BLAST search to identify known miRNAs and novel -3p and -5p derived miRNAs. Read counts to tags per million counts (TPM) was used to normalize the expression levels of miRNAs. Differentially expressed miRNAs were identified by fold change ≥ 1.4 or fold change ≤ 0.71, and *p* ≤ 0.05. The raw and processed data have been deposited into the Gene Expression Omnibus database (https://www.ncbi.nlm.nih.gov/geo/) under accession number GSE129592.

### GO enrichment analysis of DEGs

The Gene Ontology resource (GO; http://geneontology.org) is the most comprehensive and widely used knowledge-base concerning the functions of genes. GO has three ontologies including biological process, molecular function and cellular component. The basic unit of GO is GO-term. GO enrichment analysis provides the GO terms significantly enriched of DEGs compared with the genome background calculated by hypergeometric test (Ashburner et al., 2000; Gene Ontology Consortium, 2018).

\begin{eqnarray*}\mathrm{P}=1-\sum _{i=0}^{m-1} \frac{ \left( {M\atop \mathrm{i}} \right) \left( {N-M\atop m-i} \right) }{ \left( {N\atop n} \right) } \end{eqnarray*}

N: The number of all genes with GO annotation; n: The number of DEGs in N; M: The number of all genes annotated to certain GO terms; m: The number of DEGs in M. GO terms with *p* ≤ 0.05 are defined as significantly enriched GO terms in DEGs.

### KEGG enrichment analysis of DEGs

The Kyoto Encyclopedia of Genes and Genomes (KEGG) (http://www.kegg.jp/) is an encyclopedia of genes and genomes, which is the major public pathway-related database. Pathway enrichment analysis identifies significantly enriched pathways in DEGs relative to the whole genome background by hypergeometric test (Kanehisa et al., 2017; Kanehisa et al., 2011). The formula is the same as that used in GO analysis. N, The number of all genes with KEGG annotation; n, The number of DEGs in N; M, The number of all genes annotated to specific pathways; m, The number of DEGs in M. Pathways with *p* ≤ 0.05 are defined as significantly enriched KEGG pathways in DEGs.

### Integrated analysis of differentially expressed miRNAs and genes

TargetScan 7.0 and Miranda software (2010 release) were used to predict the target genes of DE miRNAs. TargetScan score percentiles ≥ 50 and Miranda max free energy values ≤ -10 were the cutoff points for targets prediction. The inversely correlated DE miRNAs and DEGs from RNA-seq were used to construct miRNA-mRNA networks using Cytoscape 3.6.0 software as described previously (Pang et al., 2019).

### qRT-PCR validation of differentially expressed genes and miRNAs

To assess the reliability of the RNA-seq and small RNA sequencing, we conducted qRT-PCR validation as previously described. Briefly, total RNA of each sample was reverse transcribed to cDNA using an M-MLV GIII First-Strand Synthesis Kit (with DNase I) (Yugong Biolabs). Then, qPCR was performed using ChamQ Universal SYBR qPCR Master Mix (Vazyme, Nanjing, China) on an ABI 7500 Real-Time PCR System (Applied Biosystems, Foster City, CA, USA). The specific primers of 19 immune-associated DEGs were listed on Table 1. For miRNA validation, specific stem-loop primers were used to reverse transcribe miRNAs according to the miRNA 1st Strand cDNA Synthesis kit (Vazyme, Nanjing, China). Then, qPCR were performed using miRNA Universal SYBR qPCR Master Mix (Vazyme, Nanjing, China) with specific forward primers (F) and the universal reverse primer within the kit following the manufacturer’s protocols. The specific stem-loop primers and forward primers of the DE miRNAs were listed on Table 2. All experiments were conducted independently three times.

**Table 2:** Specific stem-loop primers and forward primers for DE miRNAs validation.

**Primers**	**Primer sequence(5′-3′)**
mmu-miR-582-5p-loop	GTCGTATCCAGTGCAGGGTCCGAGGTATTCGCACTGGATACGACGTAACT
mmu-miR-582-5p-F	GCGCGATACAGTTGTTCAACC
mmu-miR-708-3p-loop	GTCGTATCCAGTGCAGGGTCCGAGGTATTCGCACTGGATACGACCTAGAA
mmu-miR-708-3p-F	CGCGCAACTAGACTGTGAGC
mmu-miR-1197-3p-loop	GTCGTATCCAGTGCAGGGTCCGAGGTATTCGCACTGGATACGACAGAAGT
mmu-miR-1197-3p-F	GCGCGTAGGACACATGGTCT
mmu-miR-708-5p-loop	GTCGTATCCAGTGCAGGGTCCGAGGTATTCGCACTGGATACGACCCCAGC
mmu-miR-708-5p-F	GCGCGAAGGAGCTTACAATCTA
mmu-miR-582-3p-R+1-loop	GTCGTATCCAGTGCAGGGTCCGAGGTATTCGCACTGGATACGACGGTTCA
mmu-miR-582-3p-R+1-F	GCGCGTAACCTGTTGAACAAC
mmu-miR-224-5p_R+1-loop	GTCGTATCCAGTGCAGGGTCCGAGGTATTCGCACTGGATACGACAAACGG
mmu-miR-224-5p_R+1-F	CGCGCGTAAGTCACTAGTGGTT
mmu-miR-222-3p-loop	GTCGTATCCAGTGCAGGGTCCGAGGTATTCGCACTGGATACGACAGACCC
mmu-miR-222-3p-F	CGCGAGCTACATCTGGCTACT
mmu-miR-29b-3p-loop	GTCGTATCCAGTGCAGGGTCCGAGGTATTCGCACTGGATACGACAACACT
mmu-miR-29b-3p-F	CGCGTAGCACCATTTGAAATC
mmu-miR-129-2-3p-loop	GTCGTATCCAGTGCAGGGTCCGAGGTATTCGCACTGGATACGACATGCTT
mmu-miR-129-2-3p-F	CGAAGCCCTTACCCCAAA
mmu-miR-93-3p_R+1-loop	GTCGTATCCAGTGCAGGGTCCGAGGTATTCGCACTGGATACGACTCGGGA
mmu-miR-93-3p_R+1-F	GCGACTGCTGAGCTAGCACT
mmu-miR-149-5p_R-2-loop	GTCGTATCCAGTGCAGGGTCCGAGGTATTCGCACTGGATACGACGAGTGA
mmu-miR-149-5p_R-2-F	GCGTCTGGCTCCGTGTCT
Universal Reverse Primer	AGTGCAGGGTCCGAGGTATT
U6-F	CGCTTCGGCAGCACATATACTA
U6-R	CGCTTCACGAATTTGCGTGTCA

### Statistical analysis

Student’s *t*-test was used to assess statistical significance. *p* ≤ 0.05 indicated significant difference between two groups.

## Results

### Generation of NH-E6 and NH-GFP stable cell lines

**Figure 1:**
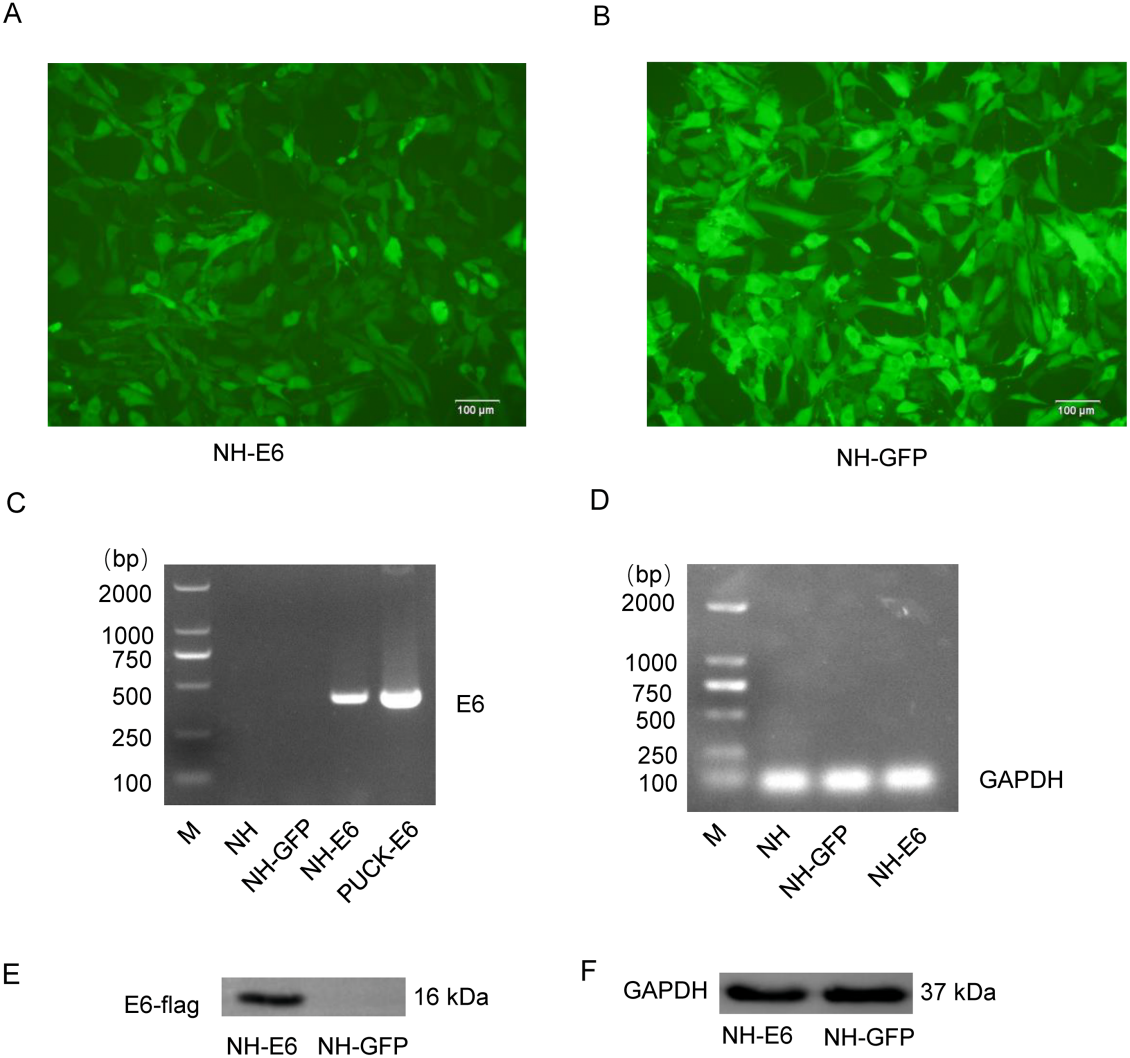
Generation of NH-E6 and NH-GFP stable cell lines. (A), (B) Images of NH-E6 and NH-GFP stable cell lines. (C), (D) BPV E6 mRNA detection by RT-PCR. The optimized E6 gene (414 bp) could only be amplified from NH-E6 stable cells and the PUCK-E6 positive plasmid but not from NH-GFP stable cells or NIH/3T3 (NH) cells. The internal control GAPDH (110 bp) could be amplified from NH sample and NH-GFP and NH-E6 stable cells. NH sample was a blank control, while PUCK-E6 plasmid was a positive control. (E), (F) BPV-13 E6 protein detection by western blot. The E6-flag fusion protein could be detected in approximately 16 kDa in NH-E6 samples while not in NH-GFP samples. The mouse GAPDH (37 kDa) was an internal control.

Sanger sequencing showed that the optimized *E6* gene had 100% amino acid identity with the wild-type gene. We observed more than 90% cells from both NH-GFP and NH-E6 group expressing bright green fluorescence. The fluorescence intensity from NH-GFP cells was a little stronger than that from NH-E6 cells (Figs. 1A, 1B). RT-PCR results indicated that the optimized *E6* gene (414 bp) could only be amplified from NH-E6 stable cells and the PUCK-E6 positive plasmid but not from NH-GFP stable cells or NIH/3T3 cells (Fig. 1C). The internal control GAPDH (110 bp) could be amplified from NIH/3T3 cells and NH-GFP and NH-E6 stable cells (Fig. 1D). The WB results indicated that a 16 kDa E6-flag protein could be detected in NH-E6 cells but not in NH-GFP cells (Fig. 1E, Fig. S3). GAPDH, a protein loading control, was detected at 37 kDa in both NH-GFP and NH-E6 samples (Fig. 1F, Fig. S3).

### Comparison of differentially expressed genes and miRNAs between NH-E6 and NH-GFP cells

**Figure 2:**
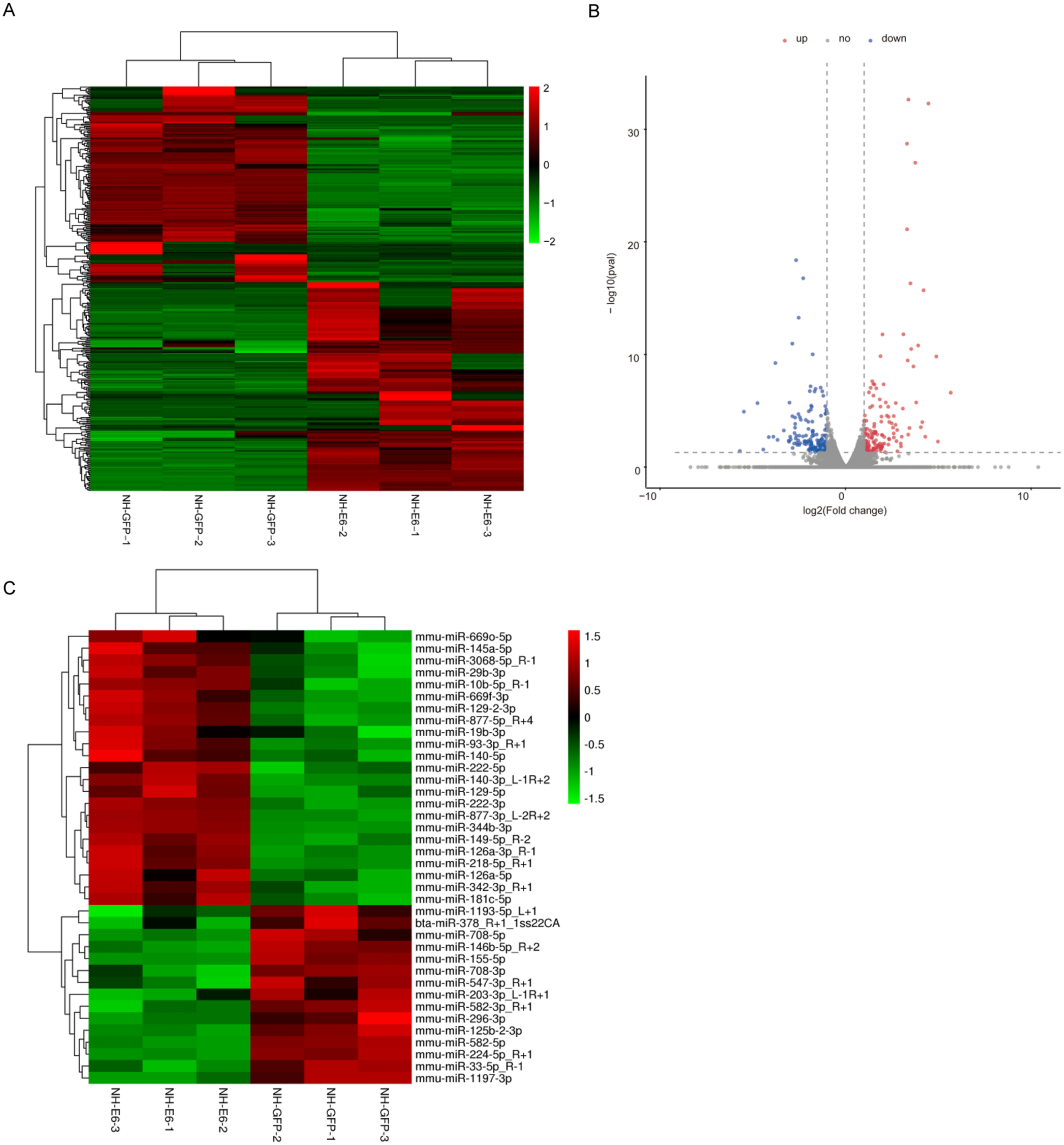
Analysis of differentially expressed genes (DEGs) and miRNAs in NH-E6 cells compared with NH-GFP cells. (A) Heatmap of the upregulated and downregulated genes. (B) Volcano plot of DEGs. Vertical lines correspond to 2-fold changes in upregulation and downregulation. Horizontal line represents *p* 0.05. The red and blue dots represent upregulated and downregulated genes, respectively, while the gray dots represent genes with no significant difference. (C) Heatmap of differentially expressed miRNAs (fold change ≥ 1.4 or fold change ≤ 0.71 and *p* ≤ 0.05 and mean TPM ≥ 50).

According to the filter criteria, fold change ≥2 or fold change ≤ 0.5 and *p* ≤ 0.05 between NH-E6 and NH-GFP samples, 350 DEGs were identified. Among these, 181 were upregulated while the other 169 were downregulated (Figs. 2A, 2B). The clustered heatmap revealed a high degree of similarity among samples within the same group and showed that the BPV-13 *E6* gene significantly altered the gene expression profile of NIH/3T3 cells.

Through small RNA sequencing, we identified 26 upregulated and 28 downregulated genes according to the cutoff, fold change ≥ 1.4 or fold change ≤ 0.71 and *p* ≤ 0.05. The upregulated and downregulated miRNAs with mean TPM ≥ 50 in NH-GFP and NH-E6 groups are presented in Tables 3 and 4. The heatmap is shown in Fig. 2C.

**Table 3:** Upregulated miRNAs in NH-E6 samples compared with NH-GFP samples (mean TPM ≥ 50).

miR_name	fold_change	*p*
*mmu-miR-877-3p_L-2R+2*	1.523507	1.45E-05
*mmu-miR-344b-3p*	1.776455	4.68E-05
*mmu-miR-222-3p*	1.439084	0.00019
*mmu-miR-149-5p_R-2*	1.92352	0.000887
*mmu-miR-140-3p_L-1R+2*	1.465576	0.001236
*mmu-miR-222-5p*	1.408298	0.004782
*mmu-miR-129-5p*	1.652894	0.004923
*mmu-miR-342-3p_R+1*	1.53254	0.004923
*mmu-miR-3068-5p_R-1*	1.547281	0.005926
*mmu-miR-29b-3p*	1.465812	0.006142
*mmu-miR-129-2-3p*	2.1222	0.006466
*mmu-miR-181c-5p*	1.515633	0.007845
*mmu-miR-126a-3p_R-1*	1.572056	0.00846
*mmu-miR-218-5p_R+1*	1.825007	0.010685
*mmu-miR-93-3p_R+1*	1.476632	0.016594
*mmu-miR-145a-5p*	1.451619	0.019651
*mmu-miR-10b-5p_R-1*	1.416059	0.02047
*mmu-miR-140-5p*	1.530396	0.028355
*mmu-miR-126a-5p*	1.448833	0.034403
*mmu-miR-19b-3p*	1.531771	0.041923
*mmu-miR-669o-5p*	1.423879	0.047297
*mmu-miR-669f-3p*	1.491341	0.012996
*mmu-miR-877-5p_R+4*	1.568047	0.001093

**Table 4:** Downregulated miRNAs in NH-E6 samples compared with NH-GFP samples (mean TPM ≥ 50).

miR_name	fold_change	*p*
*mmu-miR-582-5p*	0.490304	0.000208
*mmu-miR-146b-5p_R+2*	0.609482	0.000557
*mmu-miR-224-5p_R+1*	0.284312	0.001878
*mmu-miR-33-5p_R-1*	0.576622	0.002356
*mmu-miR-582-3p_R+1*	0.628812	0.002577
*mmu-miR-155-5p*	0.619107	0.004662
*mmu-miR-1197-3p*	0.649636	0.006704
*mmu-miR-125b-2-3p*	0.61189	0.009256
*mmu-miR-708-3p*	0.618296	0.019788
*mmu-miR-708-5p*	0.702115	0.027376
*mmu-miR-1193-5p_L+1*	0.703607	0.02951
*mmu-miR-296-3p*	0.586906	0.049283
*bta-miR-378_R+1_1ss22CA*	0.614734	0.028937
*mmu-miR-203-3p_L-1R+1*	0.367714	0.021549
*mmu-miR-547-3p_R+1*	0.501073	0.011543

### GO analysis of DEGs between NH-E6 and NH-GFP samples

**Figure 3:**
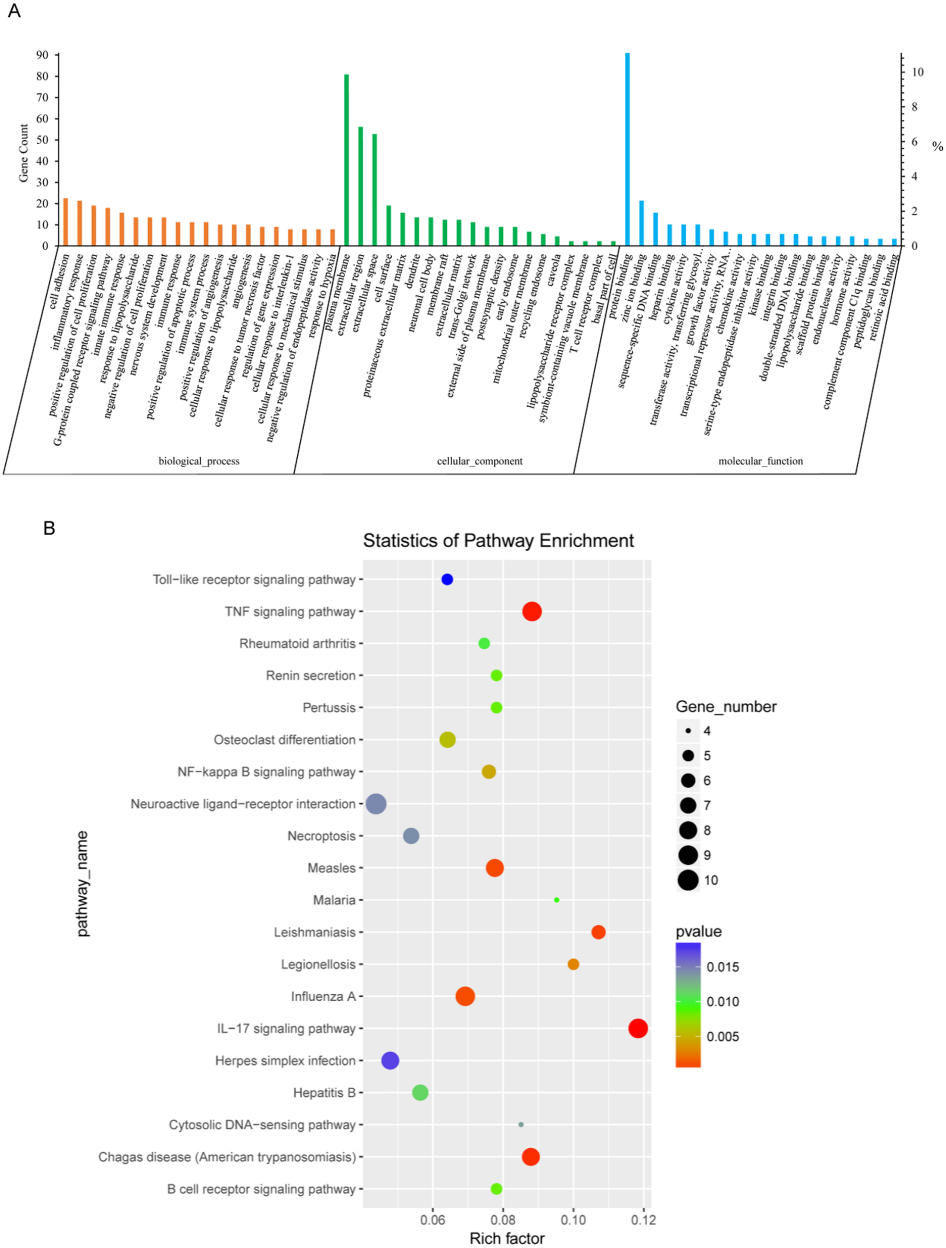
GO and KEGG analyses of differentially expressed genes in NH-E6 samples compared with NH-GFP samples. (A) GO enrichment analysis of DEGs. Top 20 GO terms (*p* ≤ 0.05) in biological processes, cellular components and molecular functions are presented. Left *Y*-axis represents the absolute gene counts enriched in the term; right *Y*-axis represents % of the genes enriched in the GO term compared to the total number of genes enriched in top 20 GO terms in three ontologies. (B) KEGG pathway enrichment analysis of DEGs. Top 20 KEGG pathways (*p* ≤ 0.05) are presented. *Y*-axis represents pathways; *X*-axis represents rich factor; (rich factor equals the ratio between the DEGs and all annotated genes enriched in the pathway); The color and size of each bubble represent enrichment significance and the number of DEGs enriched in a pathway, respectively.

Next, we conducted GO analysis of the DEGs from NH-E6 vs NH-GFP samples to gain further insight into the biological processes that are potentially regulated by these genes. There were hundreds of enriched GO terms (*P* ≤ 0.05). The top 20 significantly enriched biological processes included cell adhesion, cell cycle arrest, positive regulation of cell proliferation and apoptotic process, angiogenesis, inflammatory response, innate immune response, immune response, and immune system process (Fig. 3A). Furthermore, several significantly enriched GO terms were associated with the immune response, including inflammatory response, innate immune response, immune response, toll-like receptor signaling pathway, response to virus, chemokine-mediated signaling pathway, NF-kappaB signaling, antigen processing and presentation, as shown in Table 5.

### KEGG analysis of DEGs between NH-E6 and NH-GFP samples

Thirty-six KEGG pathways were significantly enriched (*P* ≤ 0.05). The top 20 pathways included the IL17 signaling pathway, the TNF signaling pathway, the influenza A, NF-kappa B signaling pathway, the B cell receptor signaling pathway, the cytosolic DNA-sensing pathway, and the Toll-like receptor signaling pathway (Fig. 3B). Details of several significantly enriched pathways related to the immune response are shown in Table 6.

### Construction of a miRNA-gene network

**Figure 4:**
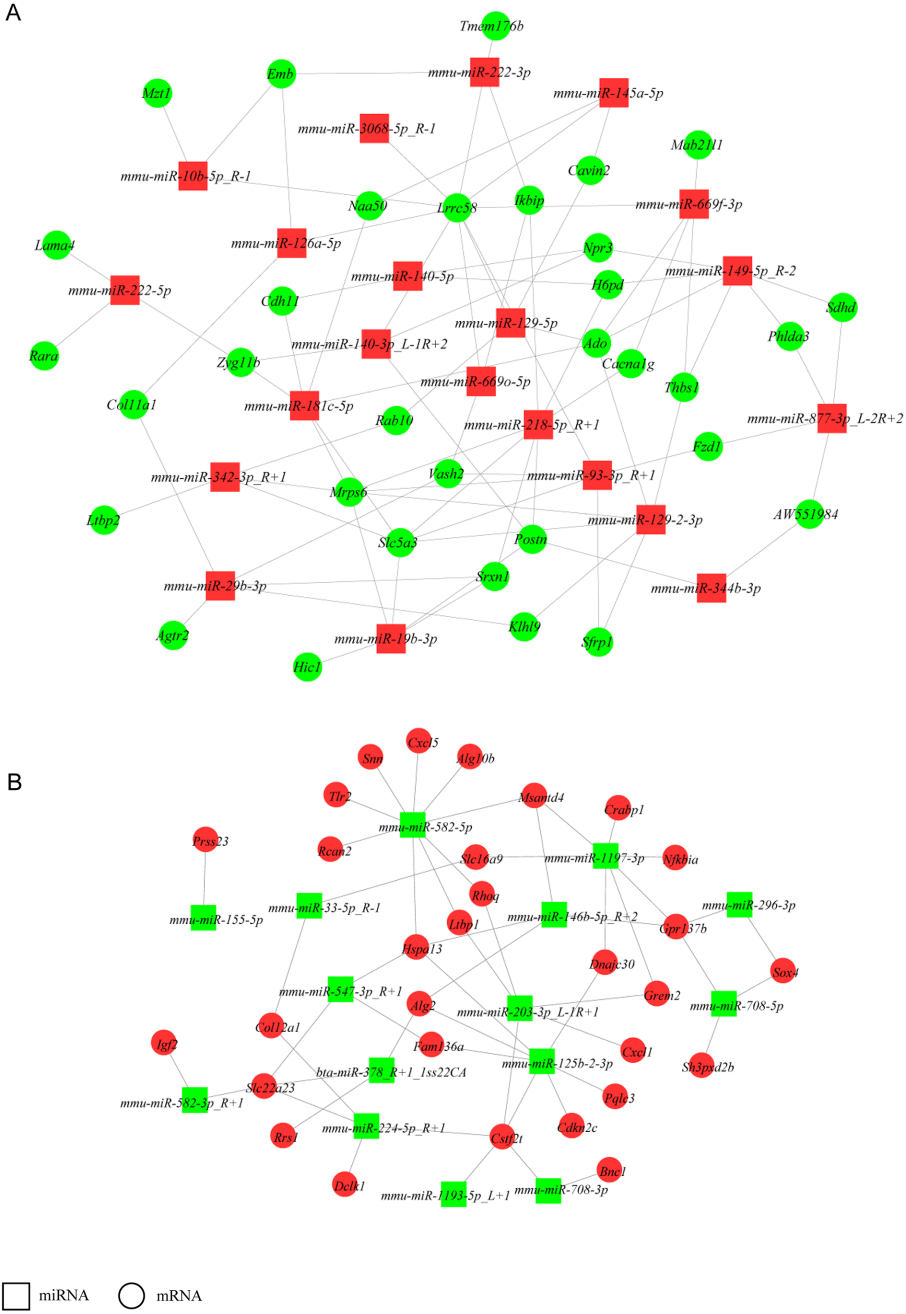
miRNA-gene regulatory networks. (A) A miRNA-gene regulatory network based on upregulated miRNAs and downregulated genes. (B) A miRNA-gene regulatory network based on downregulated miRNAs and upregulated genes. Red and green represent up- and down-regulation, respectively. Rectangle and Round represent miRNAs and genes, respectively.

To increase stringency, only DEGs with FPKM ≥5 and DE miRNAs with mean TPM ≥50 in at least one group (NH-GFP or NH-E6) were used to build a network to identify crucial miRNAs and genes in the regulatory network (Fig. 4). Thus, a total of 160 DEGs (94 upregulated, 56 downregulated) and 38 DE miRNAs (23 upregulated, 13 downregulated) were selected. We found that some miRNAs could bind several targets. For example, *mmu-miR-582-5p* potentially regulated nine targets: *Rcan2*, *Tlr2*, *Snn*, *Cxcl5*, *Alg10b*, *Msantd4*, *Rhoq*, *Ltbp1*, and *Hspa13*. Also, some miRNAs shared common targets. For instance, *Hspa13* was regulated by *mmu-miR-582-5p*, *mmu-miR-547-3p-R+1* and *mmu-miR-125b-2-3p*. These results indicate that a potential miRNA-gene regulatory network exists in BPV *E6* over-expressing NIH/3T3 cells.

### qPCR validation of DEGs and DE miRNAs

**Figure 5:**
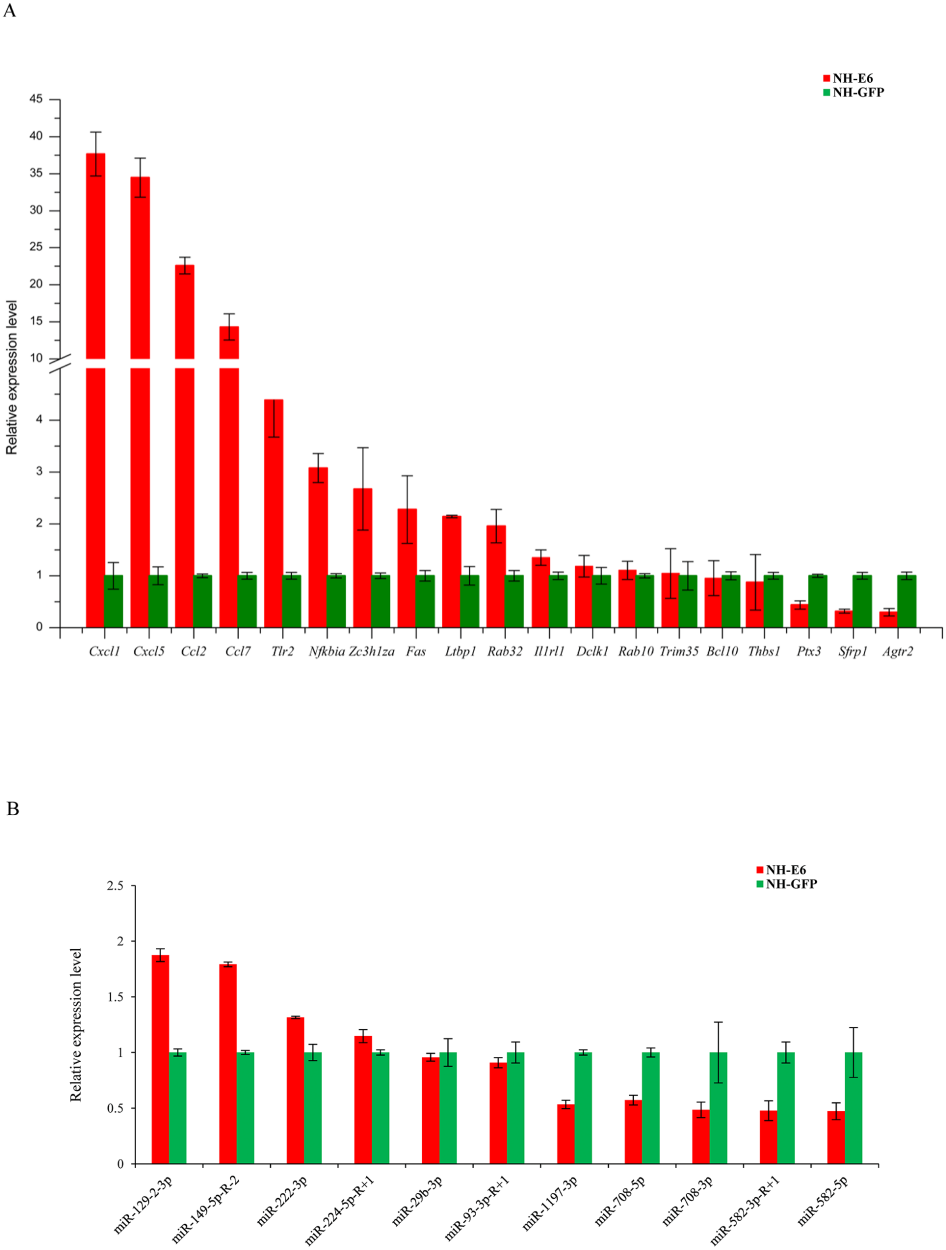
Validation of differentially expressed genes and miRNAs by qRT-PCR. (A) Validation of differentially expressed genes using qRT-PCR. Mouse GAPDH was as an internal control. (B) Validation of differentially expressed miRNAs using qRT-PCR. U6 snoRNA was as an internal control. Data from qRT-PCR assays are the means of three independent replicates, with error bars representing SD.

Based on GO and KEGG analyses, we selected 19 immune response-related DEGs (FPKM ≥5 in NH-GFP or NH-E6 groups) for qPCR validation, including 13 upregulated genes, chemokine (C-C motif) ligand 2 (*Ccl2*), chemokine (C-C motif) ligand 7 (*Ccl7*), chemokine (C-X-C motif) ligand 1 (*Cxcl1*), chemokine (C-X-C motif) ligand 5 (*Cxcl5*), toll-like receptor 2 (*Tlr2*), interleukin 1 receptor-like 1 (*Il1rl1*), nuclear factor of kappa light polypeptide gene enhancer in B cells inhibitor, alpha (*Nfkbia*), B cell leukemia/lymphoma 10 (*Bcl10*), latent transforming growth factor beta binding protein 1 (*Ltbp1*), TNF receptor superfamily member 6 (*Fas*), doublecortin-like kinase 1 (*Dclk1*), RAB32, member RAS oncogene family (*Rab32*) and zinc finger CCCH type containing 12A (*Zc3h12a*) and** six downregulated genes, angiotensin II receptor, type 2 (*Agtr2*), RAB10, member RAS oncogene family (*Rab10*), tripartite motif-containing 35 (*Trim35*), pentraxin related gene (*Ptx3*), secreted frizzled-related protein 1 (*Sfrp1*) and thrombospondin 1 (*Thbs1*). The qPCR results were consistent with the RNA-seq results for all genes except for *Bcl10*, *Trim35* and *Rab10* (Fig. 5A). Among the DE miRNAs in both NH-GFP and NH-E6 groups whose mean TPM >50, we randomly selected five upregulated miRNAs: *mmu-miR-29b-3p*, *mmu-miR-93-3p-R+1*, *mmu-miR-129-2-3p*, *mmu-miR-149-5p-R-2*, and *mmu-miR-222-3p* and six downregulated miRNAs: *mmu-miR-224-5p-R+1*, *mmu-miR-582-3p-R+1*, *mmu-miR-582-5p*, *mmu-miR-708-3p*, *mmu-miR-708-5p* and *mmu-miR-1197-3p* for qPCR validation. The qPCR results were consistent with the small RNA sequencing results for all miRNAs except for *mmu-miR-224-5p-R+1*, *mmu-miR-29b-3p* and *mmu-miR-93-3p-R+1* (Fig. 5B).

**Table 5:** Significantly enriched GO terms associated with immune response.

**GO_ID**	**GO_Term**	**Genes**	***p***
GO:0006954	inflammatory response	*Agtr2;Aim2;C3;C4b;Ccl2;Ccl7;Cxcl10;Cxcl1;Cxcl5;Cyp26b1; Fas;Nfkbiz;Relb;Sema7a;Thbs1;Tlr2;Tlr4;Tnfaip3;Zc3h12a*	0.00
GO:0050729	positive regulation of inflammatory response	*Il1rl1;Il33;Nfkbia;Ptger4;Tlr2;Tlr4*	0.00
GO:0045087	innate immune response	*Aim2;Bcl10;C3;C4b;Ighm;Oas3;Ptx3;Relb;Syk;Tgtp1; Gm12185;Tlr2;Tlr4;Trem2;Trim35*	0.00
GO:0006955	immune response	*Ccl2;Ccl7;Cxcl10;Cxcl1;Cxcl5;Fas;Oas3;Ptger4; Tgtp1;Gm12185;Thbs1*	0.00
GO:0070098	chemokine-mediated signaling pathway	*Ccl2;Ccl7;Cxcl10;Cxcl1;Cxcl5*	0.00
GO:0019882	antigen processing and presentation	*Rab10;Rab32;Relb*	0.01
GO:0007249	I-kappaB kinase/NF-kappaB signaling	*Bcl10;Nfkbia;Relb*	0.02
GO:0002224	toll-like receptor signaling pathway	*Tlr2;Tlr4*	0.03
GO:0043065	positive regulation of apoptotic process	*Bcl10;Creb1;Fas;Gadd45a;Mmp9;Phlda3; Sfrp1;Sox4;Tnfaip8;Trim35*	0.03
GO:0009615	response to virus	*Cxcl10;Dclk1;Oas3;Tgtp1;Gm12185*	0.04
GO:0002376	immune system process	*Aim2;Bcl10;C3;C4b;Oas3;Syk;Tgtp1; Gm12185;Tlr2;Tlr4;Zc3h12a*	0.04

**Table 6:** Significantly enriched KEGG pathways associated with immune response.

**pathway_id**	**pathway_name**	**Genes**	***p***
ko04657	IL-17 signaling pathway	*Ccl2;Ccl7;Cxcl10; Cxcl1;Cxcl5;Fos;Mmp9;Nfkbia;Tnfaip3*	0.00
ko04668	TNF signaling pathway	*Ccl2;Creb1;Cxcl10; Cxcl1;Fas;Fos;Mmp9;Nfkbia;Tnfaip3*	0.00
ko04064	NF-kappa B signaling pathway	*Bcl10;Nfkbia; Relb;Syk;Tlr4;Tnfaip3*	0.00
ko04620	Toll-like receptor signaling pathway	*Cxcl10;Fos;Nfkbia; Tlr2;Tlr4*	0.02
ko04621	NOD-like receptor signaling pathway	*Aim2;Ccl2;Cxcl1; Nfkbia;Oas3;Tlr4;Tnfaip3*	0.02
ko04062	Chemokine signaling pathway	*Ccl2;Ccl7;Cxcl10;Cxcl1;Cxcl5;Elmo1;Nfkbia*	0.03
ko04060	Cytokine-cytokine receptor interaction	*Ccl2;Ccl7;Cxcl10;Cxcl1;Cxcl5;Fas;Ifngr2;Vegfc*	0.04

## Discussion

The effect of *E6* oncogene expression on miRNA and mRNA expression profiles in NIH/3T3 cells was unknown. Therefore, in this study, we focused on the BPV *E6* oncogene. The RNA sequencing analysis identified 350 DEGs in BPV *E6*-overexpressing NIH/3T3 cells compared with negative control cells. To investigate the major biological processes in which the DEGs were involved, we conducted GO analysis of the DEGs. Some significantly enriched immune-associated biological processes and corresponding immune associated genes are presented in Table 5. The expression of both *Cxcl5* and *Ccl7* increased more than 10-fold in the BPV *E6* over-expressing NIH/3T3 cells compared with control cells. These results are consistent with those of a previous study in which gene expression profiles were used to identify candidate genes involved in the pathogenesis of equine skin tumors induced by BPV-1 infection (Yuan et al., 2008). Yuan et al. found that several genes enriched in inflammation and immunity, apoptosis and RNA transcription/metabolism were dysregulated; in particular, the expression of *Cxcl5* and *Ccl7* was significantly increased in BPV-1-transformed EqPalF cells. The Toll-like receptor (TLR) gene family plays a crucial role in recognizing pathogen-associated molecular patterns (PAMPs), which are conserved pathogen structures and ideal targets for recognition by the innate immune system (Bowie & Haga, 2005; Eric et al., 2007). TLR2 can recognize herpes simplex virus type 1 virions and mediate the release of inflammatory cytokines (Kurt-Jones et al., 2004), while Epstein-Barr virus induces Monocyte Chemotactic Protein 1 (MCP-1, also named CCL2) secretion by human monocytes through TLR2 (Eric et al., 2007). In our study, *Tlr2* and *Ccl2* were significantly upregulated in response to BPV-13 *E6* expression in NIH/3TR3 cells.

Accumulating evidence indicates that miRNAs play important roles in response to viral infection by directly targeting the viral genome or cellular genes (Fu et al., 2017; Ouyang et al., 2018; Slonchak et al., 2015). Terron-Canedo et al. (2017) used miRNA microarray analysis to investigate differentially expressed miRNAs in bovine papillomavirus type 1-transformed equine fibroblasts (EqPalF) compared with control cells. They found 206 differentially expressed mature miRNAs (*p* < 0.05, 144 downregulated, 62 upregulated) in EqPalFs transformed by the BPV-1 genome. The expression of six DE miRNAs (*let-7b*, *miR-17*/*23b*/*132*/*143*/*181a*) was confirmed by qPCR. This showed that miRNAs are involved in equine sarcoids and cell transformation. Previous studies reported that E6 oncoprotein from high-risk human papillomavirus type 16 (HPV16) or 18 (HPV18) inhibited the expression of tumor-suppressive miR-34a by degradation of p53, resulting in cell proliferation. HPV16 E6 also downregulated the expression of miR-23b, increased the expression of Urokinase-type plasminogen activator (uPA) and thus induced the migration of human cervical carcinoma SiHa and CaSki cells (Wang et al., 2009; Au Yeung et al., 2011). To explore miRNA changes in NIH/3T3 cells in response to BPV-13 *E6* expression, we performed small RNA sequencing. Based on the filtering criteria, fold change ≥ 1.4 or fold change ≤ 0.71, *p* ≤ 0.05 and mean TPM ≥50 between NH-E6 samples and NH-GFP controls, we identified 23 upregulated and 15 downregulated miRNAs. The dysregulated miRNAs might participate in the biological process in response to BPV infection or in response to BPV *E6*. To investigate the potential functions of the differentially expressed miRNAs, a miRNA-gene regulatory network was constructed. Interestingly, we found that *mmu-miR-582-5p* was predicted to bind to *Tlr2* and *Cxcl5*, which play crucial roles in the immune response to virus or other pathogens. Also, *mmu-miR-129-2-3p* potentially binds to *Thbs1* and *Sfrp1* and *Sfrp1* suppresses hepatoma cell growth through the Wnt signaling pathway (Yu-Lueng et al., 2010). Also, downregulation of *Srfp1* activates the Wnt pathway and contributes to cervical cancer progression (Ming-Tzeung et al., 2009). These findings indicate that *mmu-miR-129-2-3p* might participate in the transformation process by targeting *Sfrp1*, although the underlying mechanism remains unclear. The miRNA-gene network will be useful for delineating the complex cellular response to BPV *E6* expression and may provide new insights into the functions of BPV *E6*.

Although we identified several differentially expressed genes associated with the immune response and constructed a potential miRNA-gene regulatory network following BPV *E6* expression, the interactions between the miRNAs and their target genes as well as their potential functions in response to BPV *E6* remain to be further studied.

## Conclusions

In the present study, RNA sequencing and small RNA sequencing were used to explore changes in mRNAs and miRNAs in *E6* over-expressing NIH/3T3 cells compared with control cells. A total of 350 genes (181 upregulated and 169 downregulated) and 54 miRNAs (26 upregulated and 28 downregulated) were differentially expressed following *E6* expression. The major significantly enriched GO terms in “biological process” were inflammatory response, innate immune response, immune response, immune system process, positive regulation of apoptotic process, and cell adhesion. A potential miRNA-gene regulatory network was constructed. Upregulation of 12 genes: *Ccl2*, *Ccl7*, *Cxcl1*, *Cxcl5*, *Tlr2*, *Nfkbia*, *Fas*, *Il1rl1*, *Ltbp1*, *Rab32* and *Zc3h12a*, *Dclk1* and downregulation of four genes, *Agtr2*, *Ptx3*, *Sfrp1* and *Thbs1*,** were confirmed by qPCR. *Ccl2*, *Ccl7*, *Cxcl1* and *Cxcl5* were upregulated more than ten-fold in NH-E6 cells compared with NH-GFP controls. Upregulation of three miRNAs, *mmu-miR-129-2-3p*, *mmu-miR-149-5p-R-2* and *mmu-miR-222-3p*, and downregulation of five miRNAs, *mmu-miR-582-3p-R+1*, *mmu-miR-582-5p*, *mmu-miR-708-3p*, *mmu-miR-708-5p* and *mmu-miR-1197-3p*, were confirmed by qPCR. Our study investigated the differential expression of mRNAs and miRNAs in NIH/3T3 cells in response to BPV *E6* expression and provides new insights into the functions of the BPV *E6* oncogene.

##  Supplemental Information

10.7717/peerj.7442/supp-1 Figure S1 Alignment analysis of the original E6 from BPV-1, -2,-13 (Hainan strain) and the optimized BPV E6

10.7717/peerj.7442/supp-2 Figure S2 The lentiviral plasmid map

10.7717/peerj.7442/supp-3 Figure S3 BPV-13 E6 protein detection by western blot The E6-flag fusion protein could be detected in approximately 16 kDa in NH-E6 samples while not in NH-GFP samples. The mouse GAPDH (37 kDa) was an internal control. M: prestained protein ladder 26619 (Thermo Fisher Scientific).

## Additional Information and Declarations

### Data Availability

The following information was supplied regarding data availability: Data is available at Gene Expression Omnibus database: GSE129592 and GSE129593.
