# Supplementary material for: Integrated mRNA and miRNA profiling in NIH/3T3 cells in response to bovine papillomavirus E6 gene expression
Source: PeerJ. 2019 Aug 2;7:e7442. doi: 10.7717/peerj.7442 (PMC6681795; doi:10.7717/peerj.7442)
Supplement: Figure S1 [file peerj-07-7442-s001.pdf]

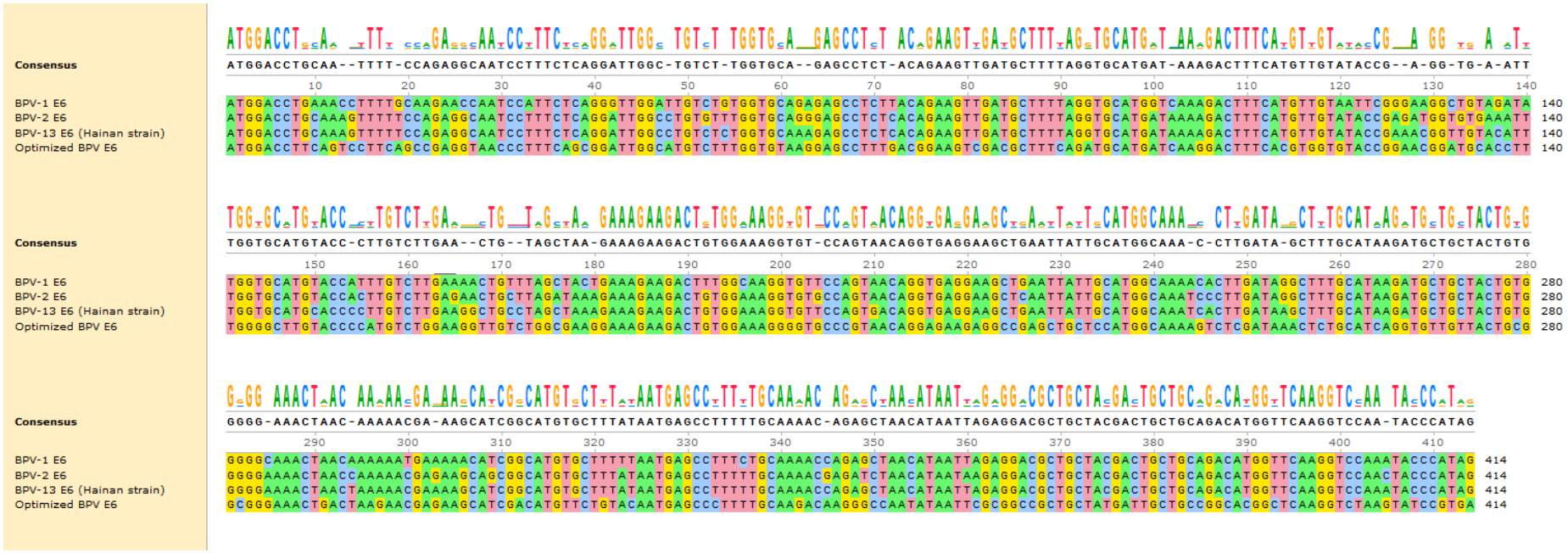
Consensus
ATGGACCTGCAA--TTTT-CCAGAGGCAATCCTTTCTCAGGATTGGC-TGTCT-TGGTGCA--GAGCCTCT-ACAGAAGTTGATGCTTTTAGGTGCATGAT-AAAGACTTTCATGTTGTATACCG--A-GG-TG-A-ATT
10 20 30 40 50 60 70 80 90 100 110 120 130 140
BPV-1 E6
BPV-2 E6
BPV-13 E6 (Hainan strain)
Optimized BPV E6
ATGGACCTGAAACCTTTTGCAAGAACCAATCCATTCTCAGGGTTGGATTGTCTGTGGTGCAGAGAGCCTCTTACAGAAGTTGATGCTTTTAGGTGCATGGTCAAAGACTTTCATGTTGTAATTCGGGAAGGCTGTAGATA 140
ATGGACCTGCAAAGTTTTTCCAGAGGCAATCCTTTCTCAGGATTGGCCTGTGTTTGGTGCAGGGAGCCTCTCACAGAAGTTGATGCTTTTAGGTGCATGATAAAAGACTTTCATGTTGTATACCGAGATGGTGTGAAATT 140
ATGGACCTGCAAAGTTTTTCCAGAGGCAATCCTTTCTCAGGATTGGCCTGTCTCTGGTGCAAAGAGCCTCTCACAGAAGTTGATGCTTTTAGGTGCATGATAAAAGACTTTCATGTTGTATACCGAAACGGTTGTACATT 140
ATGGACCTTCAGTCCTTCAGCCGAGGTAACCCTTTCAGCGGATTGGCATGTCTTTGGTGTAAGGAGCCTTTGACGGAAGTCGACGCTTTCAGATGCATGATCAAGGACTTTCACGTGGTGTACCGGAACGGATGCACCTT 140
TGGTGCATGTACC-CTTGTCTTGAA--CTG--TAGCTAA-GAAAGAAGACTGTGGAAAGGTGT-CCAGTAACAGGTGAGGAAGCTGAATTATTGCATGGCAAA-C-CTTGATA-GCTTTGCATAAGATGCTGCTACTGTG
150 160 170 180 190 200 210 220 230 240 250 260 270 280
TGGTGCATGTACCATTTGTCTTGAAAACTGTTTAGCTACTGAAAGAAGACTTTGGCAAGGTGTTCCAGTAACAGGTGAGGAAGCTGAATTATTGCATGGCAAAACACTTGATAGGCTTTGCATAAGATGCTGCTACTGTG 280
TGGTGCATGTACCACTTGTCTTGAGAACTGCTTAGATAAAGAAAGAAGACTGTGGAAAGGTGTGCCAGTAACAGGTGAGGAAGCTCAATTATTGCATGGCAAATCCCTTGATAGGCTTTGCATAAGATGCTGCTACTGTG 280
TGGTGCATGCACCCCTTGTCTTGAAGGCTGCCTAGCTAAAGAAAGAAGACTGTGGAAAGGTGTTCCAGTGACAGGTGAGGAAGCTGAATTATTGCATGGCAAATCACTTGATAAGCTTTGCATAAGATGCTGCTACTGTG 280
TGGGGCTTGTACCCCATGTCTGGAAGGTTGTCTGGCGAAGGAAAGAAGACTGTGGAAAGGGGTGCCCGTAACAGGAGAAGAGGCCGAGCTGCTCCATGGCAAAAGTCTCGATAAACTCTGCATCAGGTGTTGTTACTGCG 280
GGGG-AAACTAAC-AAAAACGA-AAGCATCGGCATGTGCTTTATAATGAGCCTTTTTGCAAAAC-AGAGCTAACATAATTAGAGGACGCTGCTACGACTGCTGCAGACATGGTTCAAGGTCCAA-TACCCATAG
290 300 310 320 330 340 350 360 370 380 390 400 410
BPV-1 E6
BPV-2 E6
BPV-13 E6 (Hainan strain)
Optimized BPV E6
GGGGCAAACTAACAAAAAATGAAAAACATCGGCATGTGCTTTTTAATGAGCCTTTCTGCAAAACCAGAGCTAACATAATTAGAGGACGCTGCTACGACTGCTGCAGACATGGTTCAAGGTCCAAATACCCATAG 414
GGGGAAAACTAACCAAAAACGAGAAGCAGCGGCATGTGCTTTATAATGAGCCTTTTTGCAAAACGAGATCTAACATAATAAGAGGACGCTGCTACGACTGCTGCAGACATGGTTCAAGGTCCAACTACCCATAG 414
GGGGAAAACTAACTAAAAACGAAAAGCATCGGCATGTGCTTTATAATGAGCCTTTTTGCAAAACCAGAGCTAACATAATTAGAGGACGCTGCTACGACTGCTGCAGACATGGTTCAAGGTCCAAATACCCATAG 414
GCGGGAAACTGACTAAGAACGAGAAGCATCGACATGTTCTGTACAATGAGCCCTTTTGCAAGACAAGGGCCAATATAATTCGCGGCCGCTGCTATGATTGCTGCCGGCACGGCTCAAGGTCTAAGTATCCGTGA 414
