# Supplementary figures and images for: Integrated mRNA and miRNA profiling in NIH/3T3 cells in response to bovine papillomavirus E6 gene expression
Source: PeerJ. 2019 Aug 2;7:e7442. doi: 10.7717/peerj.7442 (PMC6681795; doi:10.7717/peerj.7442)

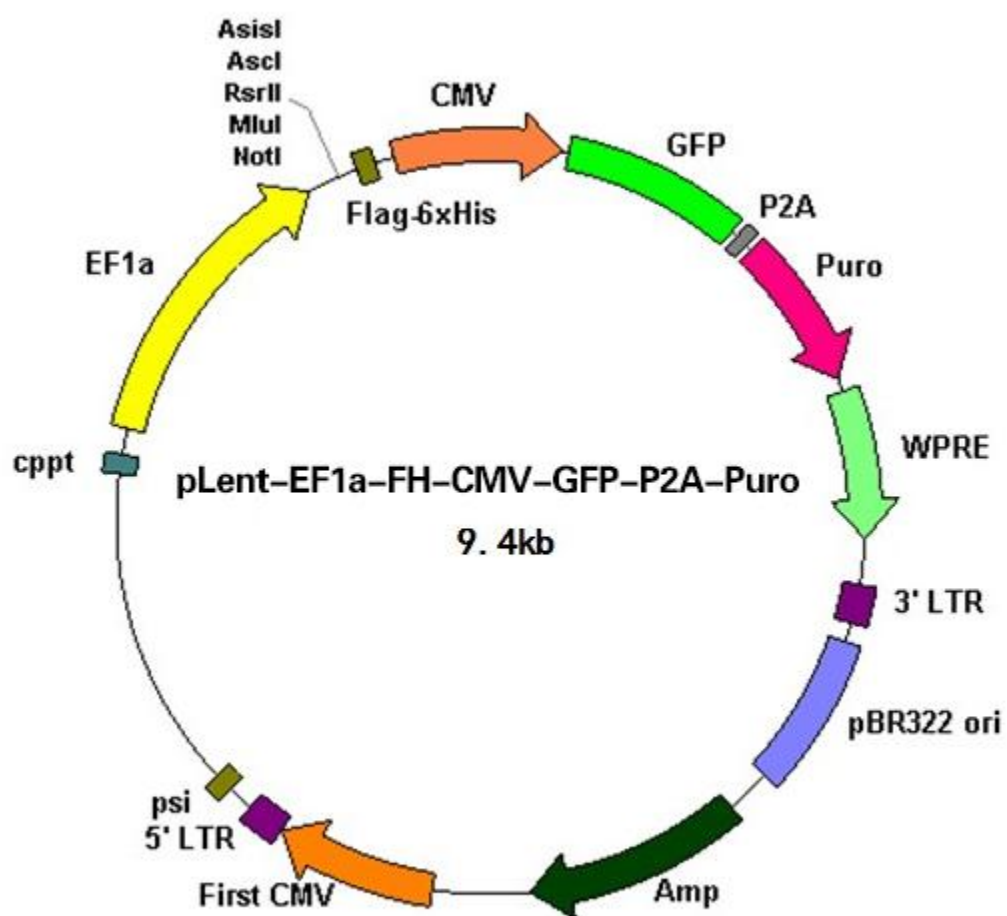

Supplement: Figure S2 [file peerj-07-7442-s002.pdf]

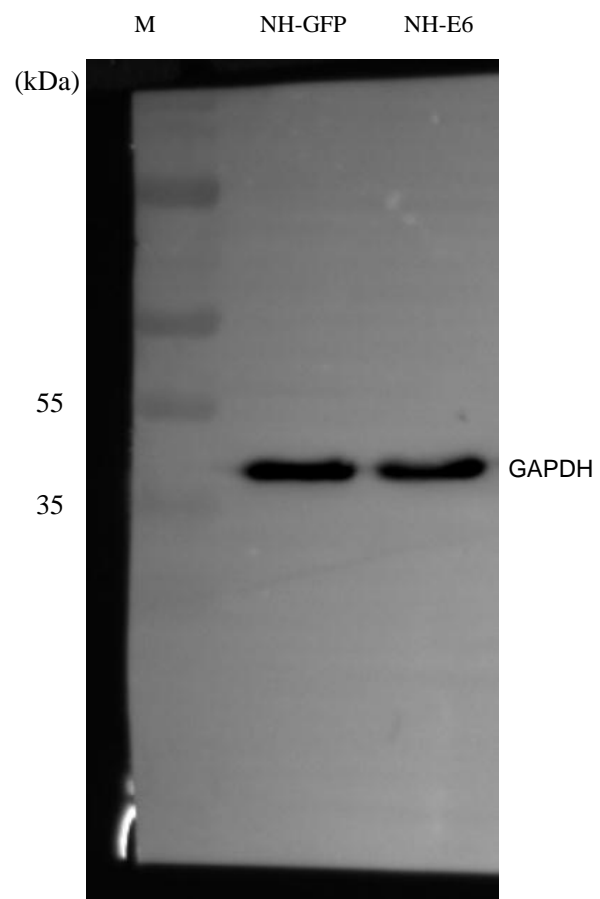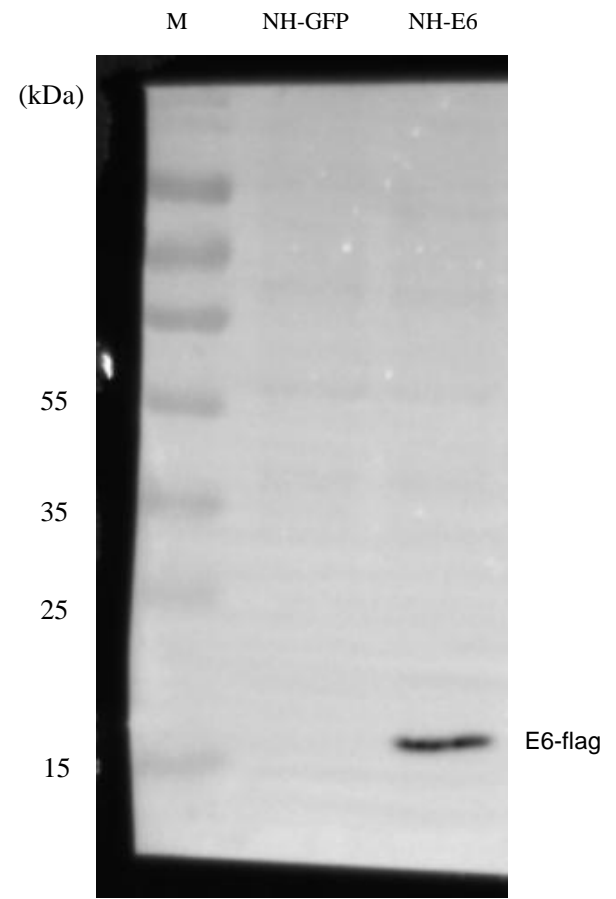

Supplement: Figure S3 — The E6-flag fusion protein could be detected in approximately 16 kDa in NH-E6 samples while not in NH-GFP samples. The mouse GAPDH (37 kDa) was an internal control. M: prestained protein ladder 26619 (Thermo Fisher Scientific). [file peerj-07-7442-s003.pdf]
